# Incidental detection of adrenal schwannoma: a case report

**DOI:** 10.1186/s13256-026-06368-2

**Published:** 2026-07-15

**Authors:** Ensiyeh Bahadoran, Freidoon Solhjoo, Samin Khoei, Afsaneh Yakhforoshha, Fatemeh SamieeRad

**Affiliations:** 1 Cellular and Molecular Research Center, Research Institute for Prevention of Non- Communicable Disease, Qazvin University of Medical Sciences, Qazvin, Iran; 2 Department of Pathobiology, School of Medicine, Faculty of Medical School, Qazvin University of Medical Sciences, Kowsar Medical and Educational Center, Taleghani St., Qazvin, 3416513176 Iran; 3 Department of Diagnostic Radiology and Nuclear Medicine, Rush University Medical Center, Chicago, IL USA; 4 Department of Radiology, Tehran University of Medical Sciences, Tehran, Iran; 5 School of Medicine, Qazvin University of Medical Sciences, Qazvin, Iran

**Keywords:** Adrenal gland neoplasms, Schwannoma, Peripheral nerve sheath tumors, Verocay bodies, Case report

## Abstract

**Background:**

Adrenal schwannomas (AS) are rare benign peripheral nerve sheath tumors that originate from Schwann cells. They are often asymptomatic and discovered incidentally on imaging for unrelated conditions. Owing to its rarity and nonspecific clinical presentation, AS can be misdiagnosed as other adrenal neoplasms, necessitating radiological and histopathological evaluation for accurate diagnosis.

**Case presentation:**

We report the case of a 53-year-old Iranian man with a history of hypertension and fatty liver disease, who presented with mild abdominal pain. Imaging studies revealed a large, well-circumscribed, heterogeneous adrenal lesion with cystic changes and calcifications, raising suspicion of an adrenal neoplasm. Laboratory investigations ruled out functional adrenal tumors. The patient underwent laparotomy and left adrenalectomy, and histopathological examination revealed a well-encapsulated tumor with spindle cell proliferation, nuclear palisading (Verocay bodies), and hyalinized blood vessels. To further confirm the diagnosis, immunohistochemical (IHC) staining was performed, which demonstrated positivity for S-100 protein, consistent with AS.

**Conclusions:**

This case highlights the importance of considering AS in the differential diagnosis of adrenal masses. Histopathological and IHC remain the gold standard for definitive diagnosis. Complete surgical resection is considered curative, and the long-term prognosis is excellent.

## Introduction

Peripheral nerve sheath tumors include a range of distinct clinicopathologic entities, from benign growths, such as schwannomas, to aggressive malignancies known as malignant peripheral nerve sheath tumors (MPNSTs) [1]. Schwannomas are well-defined, benign tumors that are typically attached to peripheral nerves. Although the majority of cases are sporadic, some are linked to familial tumor syndromes, such as neurofibromatosis type 2, schwannomatosis, or Carney's complex. Moreover, they can appear sporadically after radiation exposure [2, 3]. Histologically, schwannomas are divided into several subtypes, including melanotic, cellular, plexiform, epithelioid, and conventional schwannomas [6].

Schwannomas most frequently occur in the head, neck, mediastinum, retroperitoneum, and extremities [7]. Less than 1% of adrenal tumors are schwannomas [8]. AS originate from Schwann cells associated with the sympathetic trunk, vagus nerve, and phrenic nerve, which supply the adrenal glands [9]. Uncommon adrenal neoplasms, including ganglioneuroma, ganglioneuroblastoma, neuroblastoma, schwannoma, and malignant peripheral nerve sheath tumors, can develop independently or coexist with pheochromocytoma as composite tumors [10]. ASs are typically slow-growing and benign, though malignant cases can occur [5].

Patients with AS are typically asymptomatic and are discovered incidentally [11]. Because most AS present as adrenal incidentalomas, accurate preoperative diagnosis remains challenging [12]. Incidental adrenal masses < 1 cm require no further investigation. Incidentalomas  ≥ 1 cm warrant further workup based on dedicated guidelines, which include a comprehensive imaging and biochemical evaluation [13]. However, as imaging thresholds are imprecise and benign and malignant lesions overlap, management should be individualized. Histopathology remains the definitive diagnostic standard [14].

Patients may experience vague symptoms like persistent abdominal pain, nausea, vomiting, or lower back discomfort due to the tumor's pressure on nearby organs. This often may lead to delayed diagnosis, allowing significant tumor growth, which complicates surgical removal and increases the risk of vascular involvement [15]. AS is thought to arise from nerve fibers supplying the adrenal medulla. Periadrenal schwannoma (PAS) is the recommended term for schwannomas appearing near the adrenal gland. To date, fewer than 200 cases of AS/PAS have been reported, making them uncommon [16].

Here, we present a rare case of AS in a 53-year-old man, which was detected incidentally during a routine fatty liver evaluation and was accompanied by mild abdominal pain. Given the rarity of AS and its typically asymptomatic nature, this case highlights the importance of considering AS in the differential diagnosis of adrenal tumors. A comprehensive evaluation, including imaging studies and histopathological examinations, is crucial for an accurate diagnosis.

## Case presentation

A 53-year-old Iranian man with a history of hypertension (HTN) and anxiety came for a routine evaluation. His regular medications included losartan 25 mg and fluoxetine 5 mg. There was no known family history of endocrine tumors or neurocutaneous syndromes. The patient has been a smoker for 30 years (15 pack-years) and also reports a 5-year history of opium use. He had a prior history of obesity, weighing 125 kg, but his weight was reduced to 100 kg through dietary modifications, with ongoing efforts to continue weight loss.

The patient had a known history of fatty liver disease and underwent an abdominal ultrasound as part of a routine checkup for fatty liver assessment. Moreover, the patient complained ofmild, non-radiating RUQ abdominal pain, which had been present for approximately1 month. The pain was not associated with nausea, vomiting, or changes in bowel habits. There was no relevant psychosocial history.

On physical examination, his vital signs were stable, with a blood pressure of 128/85 mmHg and a heart rate of 78 bpm. Abdominal examination revealed no palpable masses or tenderness, and there was no hepatosplenomegaly. No lymphadenopathy or other abnormalities were noted on systemic examination.

**Fig. 1:**
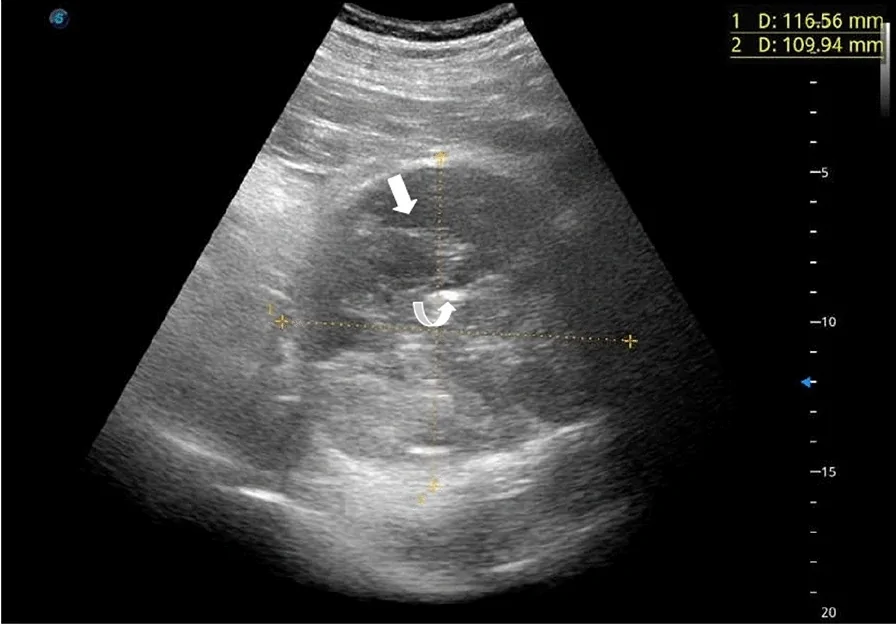
Ultrasound imaging depicts a 116*109 mm solid heterogeneous mass in the expected anatomical region of the left adrenal gland (between calipers). The mass shows internal cystic degeneration (solid arrow) and foci of coarse calcification (curved arrow)

On abdominal and pelvic ultrasonography, the shape and dimensions of the liver were normal, but its echogenicity was uniformly increased, suggesting grade 3 fatty liver. An incidentally identified 119 × 118 × 91 mm, round, heterogeneous mass containing cystic foci and internal calcification with a well-circumscribed margin was observed in the retroperitoneum adjacent to the superior ponle of the left kidney and the umbilicus of the spleen (Fig. 1).

**Fig. 2:**
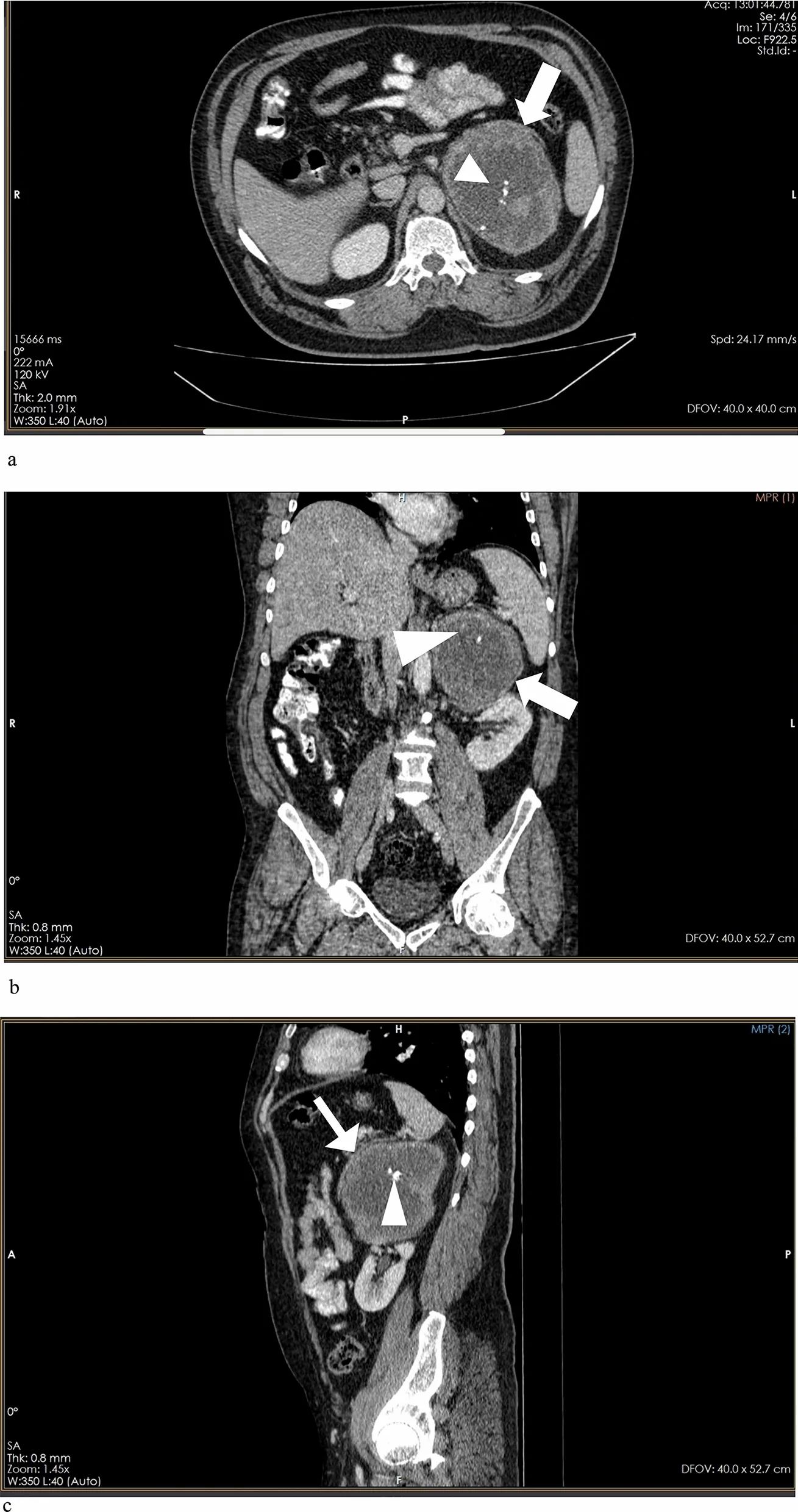
Contrast-enhanced CT scan in axial (**a**), coronal (**b**), and sagittal (**c**) planes show a sharply marginated solid-cystic mass (arrow), replacing the left adrenal gland and displacing the left kidney inferiorly. The mass contains scattered foci of coarse calcification (arrowhead). No evidence of invasion to the adjacent organs is visualized

Further examination of the origin of the lesion was recommended Regarding our institutional imaging protocol, a dual-phase (portal and delayed), contrast-enhanced CT scan of the abdomen and pelvis was performed, which demonstrated a well-circumscribed heterogeneous left adrenal mass measuring 119 × 118 × 91 mm. The lesion contained internal cystic degeneration and calcified foci. Lacking the initial non-contrast scan, the mean primary density of the lesion and the degree of enhancement couldn’t be measured. Nevertheless, no focus of macroscopic fat density was observed. No evidence of adjacent organ invasion, vascular encasement, or regional lymphadenopathy was identified (Fig. 2).

. A comprehensive hormonal evaluation including plasma-free metanephrines and a 24-h urine collection of catecholamines and metanephrines, turned out unremarkable. Additionally, a low-dose dexamethasone suppression test was performed to exclude cortisol excess. Serum aldosterone and renin levels were measured because of the patient’s history of hypertension, although they were unremarkable. Because ACC can secrete adrenal androgens, DHEA-S levels were also evaluated and found to be normal. Given the absence of hormonal hypersecretion, the lesion was classified as a nonfunctional adrenal mass.

The patient underwent a left adrenalectomy via laparotomy. Intraoperatively, a well-encapsulated 14-cm mass was identified in the left adrenal region. The tumor was carefully dissected from surrounding structures and excised en bloc along with the adrenal gland. There was no evidence of invasion into adjacent organs or major vascular structures. Estimated blood loss was approximately 150 mL, and no blood transfusion was required. No intraoperative complications were observed.

**Fig. 3:**
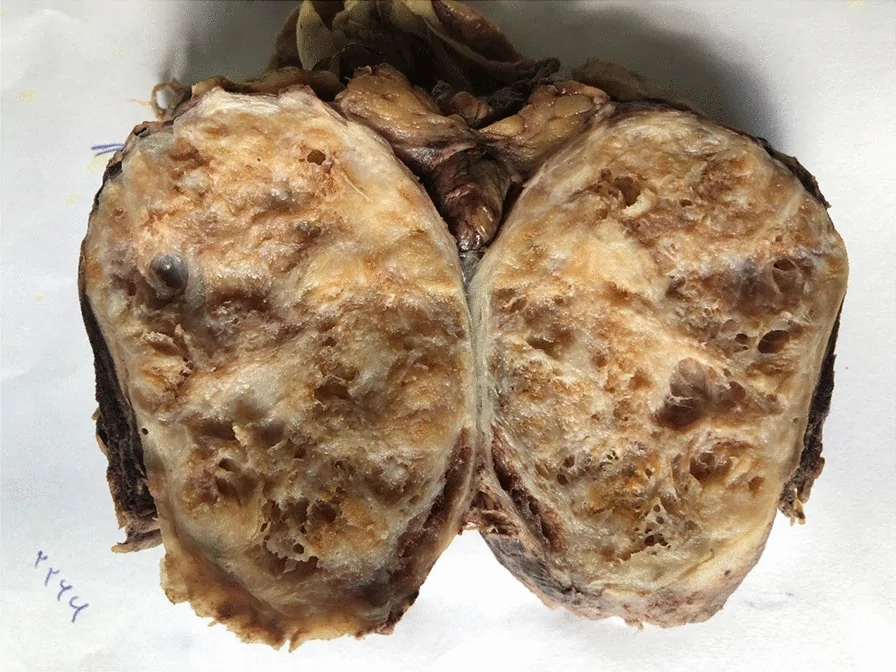
.

The specimen was then sent for histopathological evaluation. After resection of the left adrenal gland mass, macroscopic evaluation of the tumor showed an intact dark gray encapsulated mass measuring 140 × 100 × 55 mm, associated with a remnant of the adrenal gland measuring 20 × 10 × 3 mm. The mass on serial cutting showed creamy yellow soft tissue with cystic formation and hemorrhage (Fig. 3).

### Histopathological findings

**Fig. 4:**
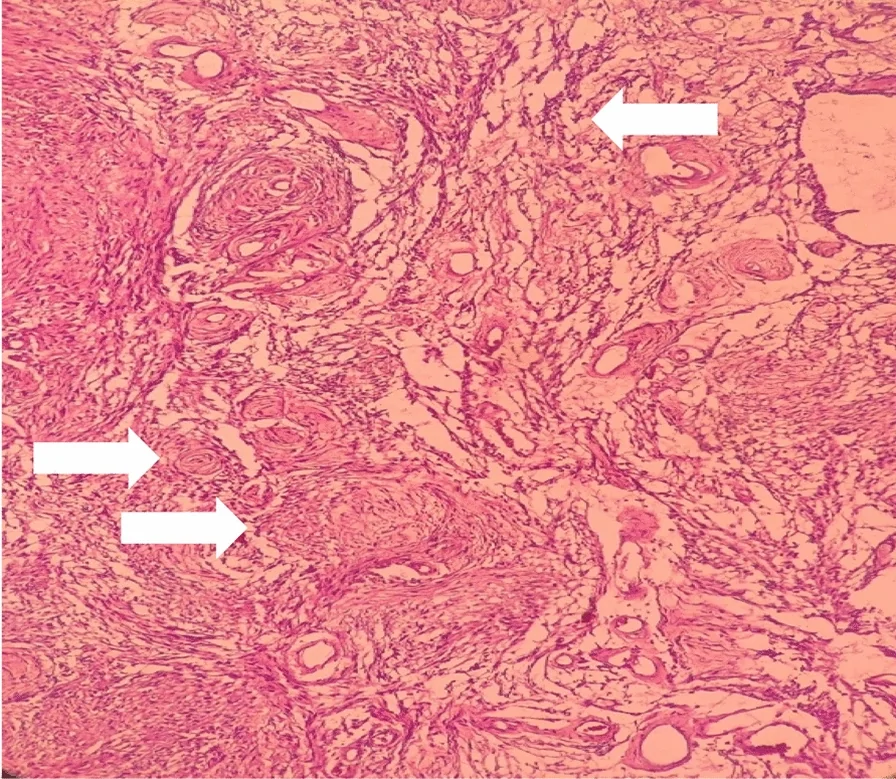
Histopathological examination of the tumor (H&E, 100 ×) shows encapsulated, well-vascularized neoplasm with two distinct patterns: cellular areas with spindle cells arranged in a palisading fashion forming Verocay bodies (left, white arrow) and regions with abundant edematous fluid (right, white arrow). Hyalinized vascular walls and large nodular collagen masses with radiating edges are also observed

**Fig. 5:**
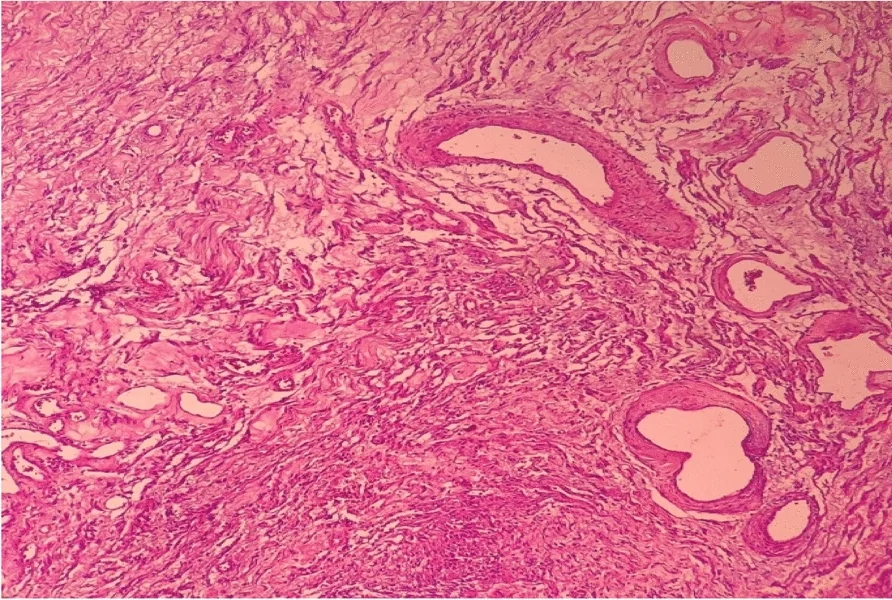
Histopathological examination of the tumor (H&E, 100 ×) showing encapsulated, well-vascularized neoplasm with two distinct patterns: cellular areas with spindle cells (lower, white arrow) and regions with abundant edematous fluid (upper, white arrow). Hyalinized vascular walls and nodular collagen masses are also observed

Microscopic evaluation revealed encapsulated, well-vascularized neoplasms in two patterns, including a quite cellular component composed of spindle cells arranged in a palisading fashion or Verocay bodies. In other areas, the tumor cells were separated by abundant edematous fluid. The mitotic activity was poor. Large nodular masses of collagen with radiating edges are also observed. Several vascular channels with hyaline-thickened walls have been observed. Based on these findings, the diagnosis of this mass was schwannoma (Fig. 4,5).

### Immunohistochemical findings

**Fig. 6:**
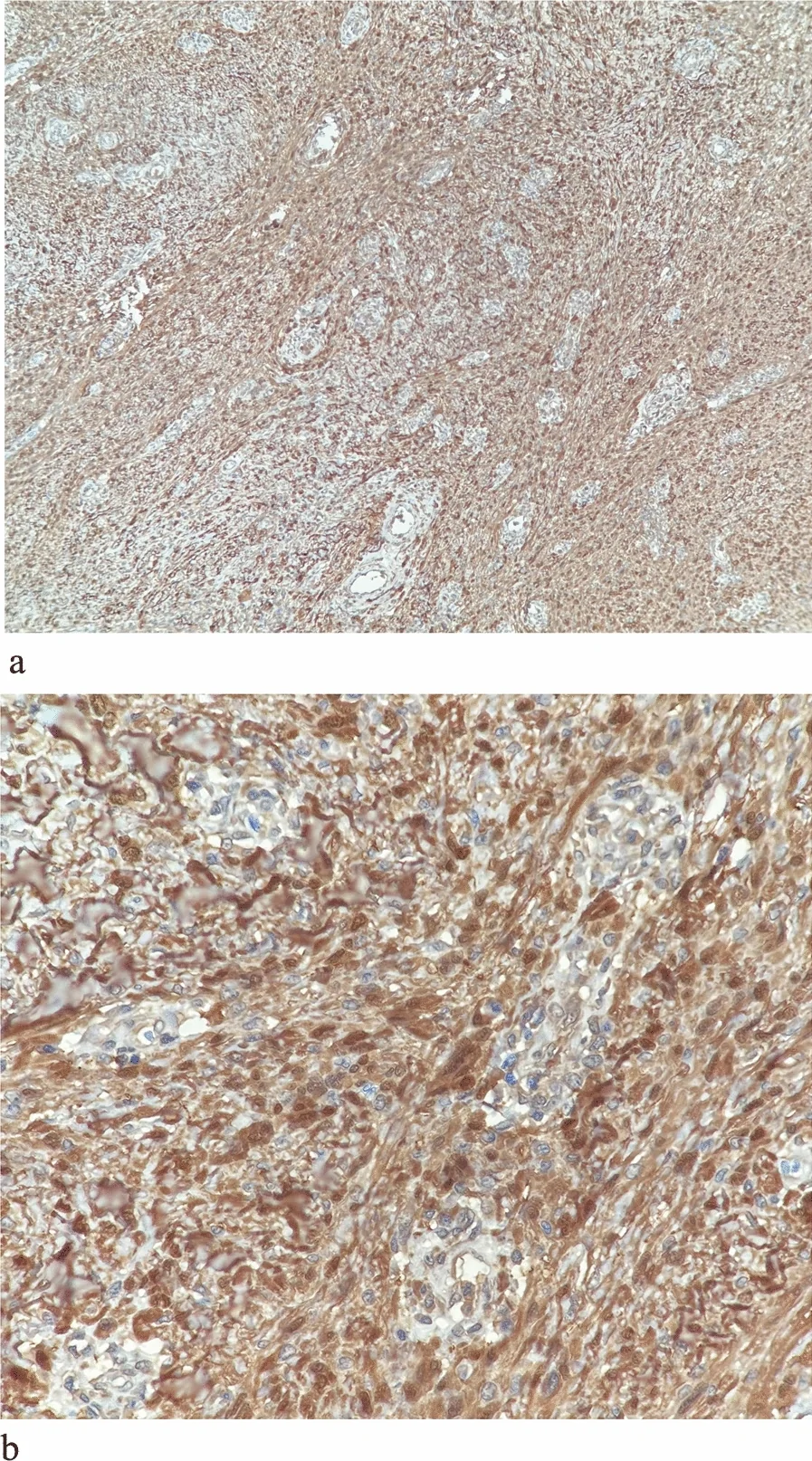

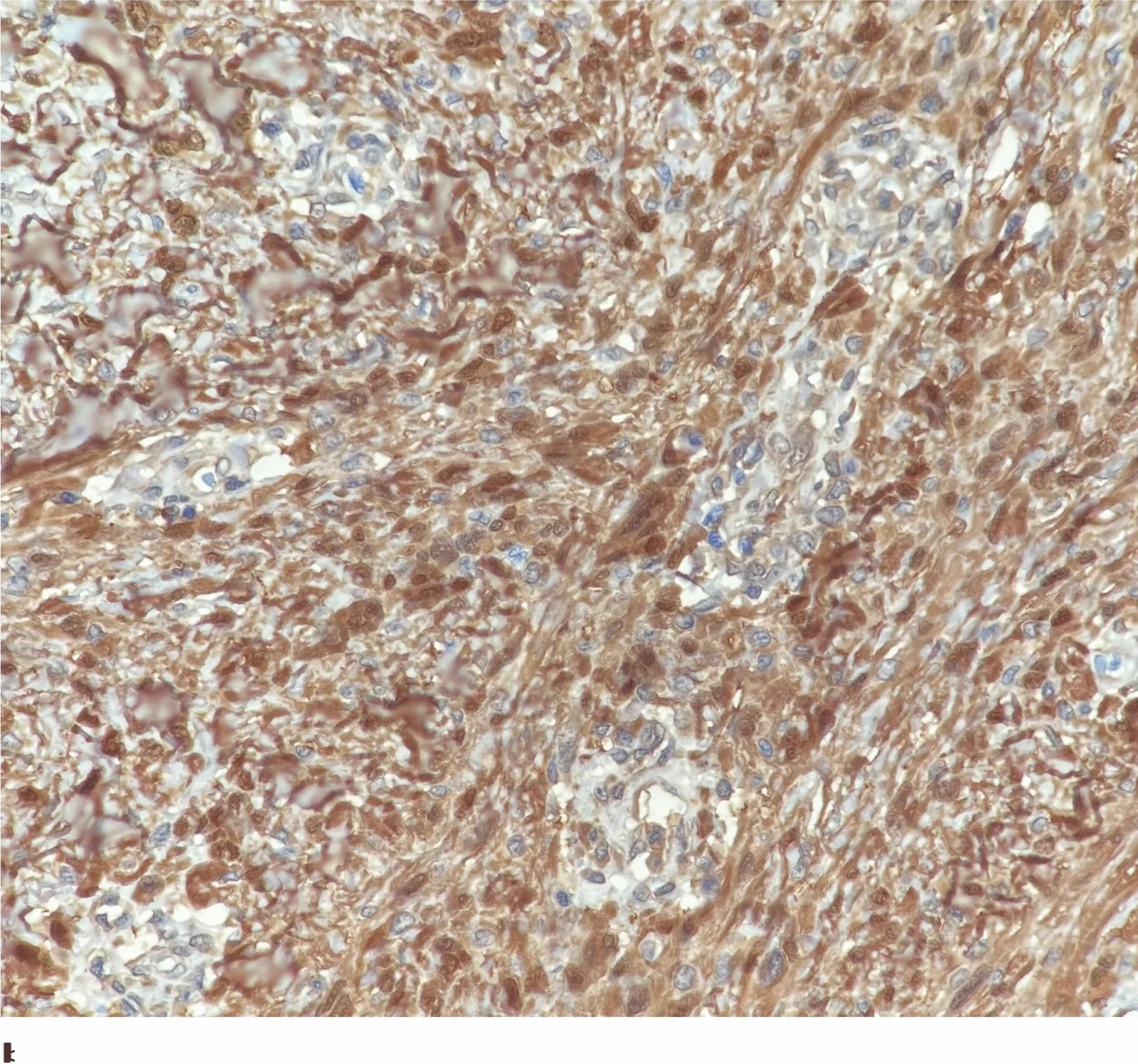
S100 IHC staining. a and b show low and high power of strong cytoplasmic and nuclear s100 IHC staining in tumor cells. (Counterstain with hematoxylin. A, *100 and B, *400)

**Fig. 7:**
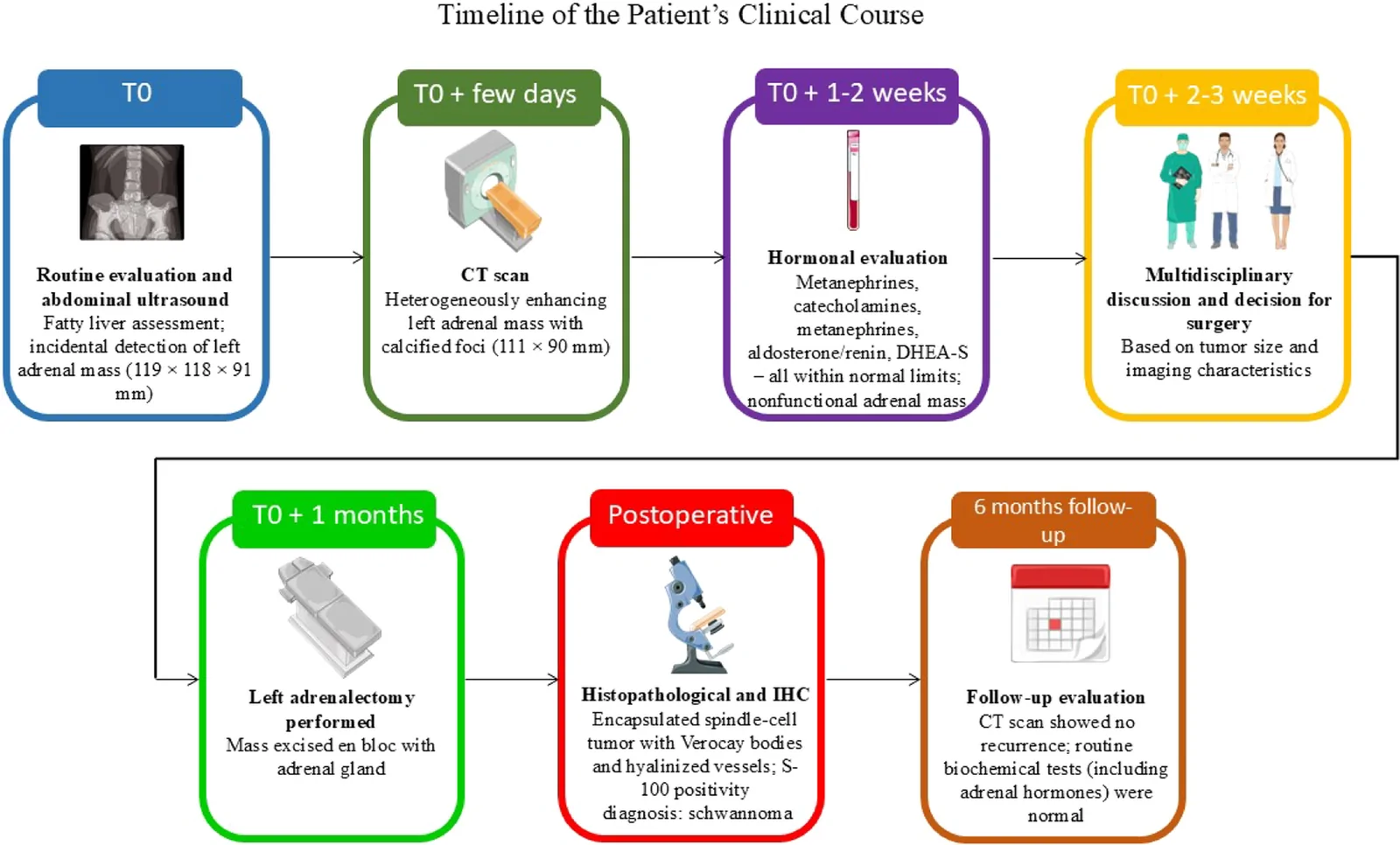
Timeline of the patient’s clinical course

To further confirm the diagnosis, immunohistochemical staining was performed, which demonstrated strong, diffuse cytoplasmic and nuclear positivity for S-100 protein, consistent with an AS (Fig. 6).The postoperative course was uneventful, and the patient was discharged on postoperative day 4. His right upper quadrant abdominal pain resolved completely within one week after surgery and did not recur during follow-up. Blood pressure remained stable on his preexisting antihypertensive regimen. Serum electrolytes, renal function tests, and morning cortisol levels were within normal limits, and there was no evidence of adrenal insufficiency. At outpatient visits conducted 3 months, and 6 months after surgery, the patient remained asymptomatic with no constitutional symptoms or abdominal complaints. Contrast-enhanced abdominopelvic CT performed at 6 months demonstrated no residual or recurrent disease. Given the benign histopathologic diagnosis and complete excision with negative margins, annual clinical and radiologic surveillance for up to 2–3 years was recommended. To better illustrate the sequence of diagnostic and therapeutic interventions, a timeline of the patient’s clinical course is provided (Fig. 7).

## Discussion

This case report presents an AS detected incidentally during a fatty liver checkup in a patient with mild abdominal pain. These findings highlight the importance of considering this rare entity in the differential diagnosis of adrenal masses.

After Verocay originally identified Schwannomas in 1908, Antonini in 1920 divided these tumors into two different histologic patterns, Antoni A and Antoni B, according to the cellular organization and architecture of the tumors [17]. Schwannomas account for 0.5% to 1.2% of benign tumors found in the retroperitoneal region[11]. AS is more commonly diagnosed in women, typically in their 40 s [18]. The size of AS ranges from 0.6 to 14 cm, and the average maximum diameter of the tumor is about 5.5 cm [11]. Although most ASs are benign and grow slowly, adrenal incidentalomas larger than 4 cm are linked to an increased risk of malignancy [9, 15]. The size of the adrenal mass and the age of the patient were consistent with those in the previous literature, although our case involved a male patient. Additionally, the lesion was benign and showed no signs of invasion or metastasis.

[19]These tumors originate from the nerve sheaths and usually compress the adjacent adrenal cortex. They seem to originate from the adrenal medulla because there is no septum surrounding the tumor [20]. Given the space in the retroperitoneal cavity, AS usually enlarges before exhibiting symptoms such as mild flank or abdominal pain [15]. Similarly, our patient also presented with mild abdominal pain.

[21]. Early-stage schwannomas are difficult to diagnose because they grow slowly and frequently show no clinical signs [22]. In a recent case series, it was found that only five of the 31 patients had symptoms, meaning that 84% of the observations were incidental [23]. Adrenal lesions incidentally detected in imaging performed for other reasons (the same scenario in our patient) warrant further evaluation only if they measure ≥ 1 cm in the short axis. As the next step, the radiologist should look for the diagnostic benign imaging features (macroscopic fat, benign calcification, mean density of ≤ 10 HU on non-contrast CT scan, lack of enhancement on post-contrast CT scan, and/or signal drop on opposed-phase magnetic resonance images). Incidentalomas ≥ 4 cm in a non-cancer patient, which lack these diagnostic benign features, should undergo surgical resection. A comprehensive biochemical assay is necessary prior to any surgery/biopsy to exclude a functional lesion. The remaining indeterminate adrenal lesions ≤ 1—< 4 cm need further evaluation by a dedicated adrenal CT scan (accompanied by measurements of absolute and relative percentage washout), follow-up, or resection depending on the individualized clinical scenario [13]. The initial differential diagnoses upon encountering this lesion included pheochromocytoma (which proved unlikely after the biochemical assay), lipid-poor adrenocortical adenoma (which is usually smaller), adrenocortical carcinoma (which remained the most probable diagnosis until after histopathology proved it wrong), and adrenal ganglioneuroma (which was unlikely regarding the significant heterogeneity of the lesion and the patient’s age). We perform a dual-phase (portal and delayed) contrast-enhanced CT scan in patients with abdominal-pelvic masses of unknown origin as the first step. As a result, we unfortunately couldn’t determine most of the diagnostic benign adrenal incidentaloma features in our patient. However, lack of macroscopic fat or benign-appearing calcification, significant heterogeneity of the lesion, and size of > 4 cm led to a final decision of surgical resection without further exposing the patient to radiation. Comparative clinicopathologic and immunohistochemical features of AS and its major differential diagnoses are provided in Table 1.

**Table 1:** Comparative clinicopathologic and immunohistochemical features of AS and its major differential diagnoses

Feature	Adrenal Schwannoma	Pheochromocytoma	Adrenocortical Carcinoma	Ganglioneuroma	Adenoma
Hormonal activity	Nonfunctional	Catecholamine-secreting	~ 40–60% are hormone-secreting	Nonfunctional	Usually nonfunctional; can be hormonally active depending on subtype
Imaging	Well-circumscribed, heterogeneous, cystic degeneration possible	Hypervascular, marked enhancement	Large, irregular, necrotic/hemorrhagic	Well-defined, homogeneous, ± calcifications	Small, homogeneous, lipid-rich
Histopathology	Antoni A/B areas, Verocay bodies	Zellballen pattern	Pleomorphism, necrosis, atypical mitoses	Mature ganglion cells in Schwannian stroma	Uniform cortical cells
Key IHC markers	S-100 + , SOX10 +	Chromogranin + , Synaptophysin +	SF-1 + , Melan-A + , Inhibin-α +	S-100 + (stroma), Synaptophysin + (ganglion cells)	SF-1 + , Melan-A + , Inhibin-α +
Biologic behavior	Benign	Behavior unpredictable; potentially malignant	Malignant	Benign	Benign

Source: Harrison’s Principles of Internal Medicine, 21st ed [33]; Goldman-Cecil Medicine, 27th ed [34]

In terms of morphology, AS is a round, encapsulated, hypovascular tumor with a uniform texture. However, calcifications and cystic or hemorrhagic degeneration have been documented to cause a heterogeneous appearance in up to 40% of instances [9]. On histopathological examination, these tumors exhibited loosely textured hypocellular regions (Antoni B areas), which alternate with hypercellular regions with spindle-shaped Schwann cells (Antoni A areas) and nuclear palisading (Verocay bodies) [2]. Histopathology provides a conclusive diagnosis of schwannoma when the tumor exhibits spindle cells with hyalinization, cystic degeneration, or rare mitoses. Schwannomas show significant S-100 antigen, vimentin, collagen IV, and laminin positivity on IHC and negative for CD 34, CD117, keratin, desmin, and smooth muscle actin (SMA). Neuroectodermal tumors are characterized by diffuse positivity for S-100 protein and distinct nuclear freeze zones known as “Verocay bodies” [24]. Calretinin helps to differentiate schwannomas from neurofibromas, as both share similar S-100 positivity [20, 25].

On an abdominal and pelvic CT scan, AS is typically seen as a heterogeneous, well-circumcised, round or oval mass with a cystic lesion, as well as sporadic calcification and septation. According to a study by Shen et al., schwannomas can be ruled out by examining the arterial phase of a CT scan that exhibits necrosis and a low level of tumor enhancement [21]. Furthermore, larger tumors (> 8–10 cm) may exhibit calcification, necrosis, hemorrhage, and cystic development because of secondary degeneration, indicating the last phase of long-term tumor progression [26]. MRI typically displays a low signal on T1-weighted images (T1WI) and a heterogeneous high signal on T2-weighted images (T2WI) [19].

Although rare, schwannomas can transform into MPNSTs, affecting their treatment and prognosis. In our case, MPNST was ruled out because of the absence of invasive features, mitotic activity, necrosis, or heterologous components [22].

Radiation and chemotherapy are ineffective for schwannoma. According to current guidelines, adrenal masses larger than 4 cm are associated with an increased risk of malignancy and should be evaluated in a risk-stratified manner, taking into account imaging characteristics and hormonal activity, with management decided in a multidisciplinary setting [22]. Robot-assisted laparoscopic surgical excision and other minimally invasive surgical techniques seem to be more appropriate for tumors smaller than 7 cm. On the other hand, laparotomy is appropriate for AS of all sizes, particularly for tumors larger than 10 cm in diameter [30]. The tumor in our case was approximately 11 cm in diameter, qualified for surgical treatment, and laparotomy was performed.

The prognosis is favorable after total surgical resection. According to Zhou et al., these patients had a good prognosis and showed no signs of recurrence following a 7–115 months of follow-up [31]. For two years, it is recommended that patients be followed up periodically using control imaging investigations, such as CT or MRI, and then once a year for three more years [32].

### Clinical practice takeaways

- AS should be considered in the differential diagnosis of large nonfunctional adrenal masses, particularly when imaging demonstrates a well-circumscribed lesion with cystic degeneration and calcifications.
- Preoperative diagnosis is difficult because imaging findings overlap with those of pheochromocytoma, ACC, ganglioneuroma, and other adrenal neoplasms.
- Comprehensive hormonal evaluation is essential to exclude functional adrenal tumors before surgery.
- Definitive diagnosis relies on histopathological examination demonstrating Antoni A and Antoni B areas, Verocay bodies, and diffuse S-100 positivity.
- Complete surgical excision is curative in most cases and is associated with an excellent long-term prognosis.

## Conclusion

ASs are rare and benign tumors that can be challenging to diagnose due to their nonspecific clinical and imaging features. This case underscores the importance of including AS in the differential diagnosis of adrenal masses, particularly when imaging reveals well-circumscribed lesions with cystic changes and calcifications. Histopathological evaluations remain the gold standard for definitive diagnosis. Surgical resection is the treatment of choice and offers an excellent prognosis with minimal risk of recurrence.

## Data Availability

Not applicable.

## Abbreviations

AS: Adrenal schwannomas
MPNSTs: Malignant peripheral nerve sheath tumors
PAS: Periadrenal schwannoma
ACC: Adrenocortical carcinoma
SMA: Smooth muscle actin
NSE: Neuron-specific enolase
